# Mapping green infrastructure and socioeconomic indicators as a public management tool: the case of the municipalities of Andalusia (Spain)

**DOI:** 10.1186/s12302-020-00418-2

**Published:** 2020-10-28

**Authors:** José Luis Caparrós Martínez, Juan Milán García, Nuria Rueda López, Jaime de Pablo Valenciano

**Affiliations:** Department of Business and Economics, Applied Economic Area, University of Almeria, Almeria, Spain

**Keywords:** Green infrastructure, Ecosystem services, Municipalities, Socioeconomic indicators, Geographic information systems, Cluster analysis

## Abstract

**Background:**

Green Infrastructure (GI) is defined as a strategically planned network of natural and semi-natural spaces that provide society, in both rural and urban areas, with a large number of goods and services of great value and economic importance such as clean air and water, carbon storage, pollination or protection against the effects of climate change. Traditionally, municipalities, like other territorial units, are characterized by a series of social and economic indicators that determine their degree of local development. The objective of this article is to identify and assess, through a system of indicators, what role urban and rural municipalities in Andalusia (Spain) play in the provision and reception of ecosystem services. To this end, Geographical Information System (GIS) techniques are used and a cluster analysis is carried out to contrast the results.

**Results:**

Rural municipalities show the largest portion of GI area in the whole region. However, they show a low socioeconomic level, with high unemployment rates.

**Conclusions:**

It can be said that the municipalities in rural areas are "ecologically" financing the entire Andalusian population. Faced with this situation, the decisions, and actions of policymakers in this region should aim at promoting measures that can restore and conserve GIs, addressing the demographic and/or socioeconomic imbalances of the region.

## Background

**Fig. 1:**
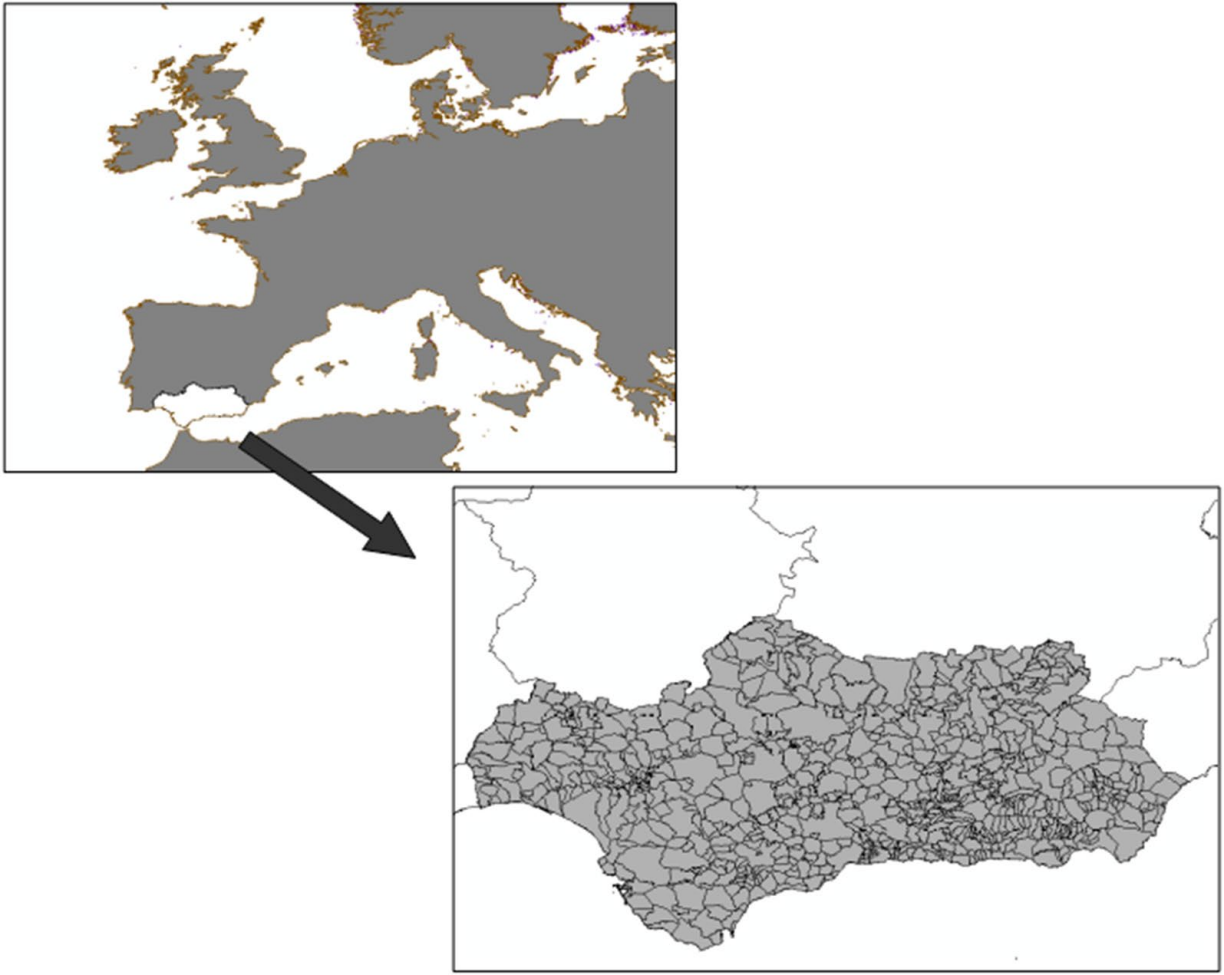
Study Area. Source: *Red de Información ambiental de Andalucía – REDIAM* (Environmental Information Network of Andalusia) (2010)

Andalusia, a southern region in Spain, is located in a temperate zone in the northern hemisphere, at a biological crossroads between two great continents, *Europe and Africa*, and between two great bodies of water, the *Mediterranean Sea and the Atlantic Ocean* (Fig. 1). This location gives it a unique biological, geological and landscape diversity, and a wide variety of rich ecosystems, ranging from arid spaces, high mountains, marshes, dunes and coastal sands, forests and countryside, among others. This privileged situation has determined that, since time immemorial, this region has been occupied by various cultures that have left their mark through a model use of natural resources [62, 63], perfectly adapted to the environmental conditions and which has favored this unique and rich biodiversity [36, 37, 57, 61]. It is hard to find in Europe a territory as populated (8,414,240 inhabitants) and extensive (87,599 km^2^ in area and 910 km of coastline) that is also so rich in natural resources or that is better conserved [26, 31, 32]. In addition, it is a territory where rural spaces have an important presence.

The region of Andalusia is part of one of the 20 most relevant biodiversity points on the planet with 28.7% of its surface listed as a natural space protected by regional, national and international regulations [37] and is key for the provision of ecosystem services. However, the sustainable interaction that has existed for centuries between nature and the human population is at risk. This is made manifest in the problems of depopulation and abandonment of traditional land uses, which in many cases, are causing a significant degradation of the ecosystems that make up the GI [5, 37];. The data are compelling. Half of the municipalities in Andalusia have lost population so far this century. Depopulation affects 389 municipalities, which represent 48.41% of Andalusia. Of these, 89.2% are rural municipalities (347/389), some of which, although small in size, suffer the greatest relative loss of population [22, 23, 25, 27].

There are exceptions to this scenario, such as the municipalities that are located in the Guadalquivir Valley and along the coast, which in recent years have seen a palpable growth in their population. At present, there are municipalities with fewer than 5,000 inhabitants that occupy 51.08% of the territory with 11.01% of the population, while large cities occupy 7.95% of the territory, concentrating 50.75% of the population [21].

Situations, such as the current COVID-19 health crisis and its spread from animals to humans can be linked to the alterations and impacts suffered by the planet's natural ecosystems [42, 67]. There is empirical evidence of the protective effect of nature and biodiversity against pathogens and infections. This capacity lies in the fact that healthy ecosystems harbor a great diversity of species that can act as hosts for pathogens, limiting the transmission of diseases, either by dilution or damping of the existing viral load. [20, 41, 44].

Although the existence of healthy ecosystems which are rich in biodiversity is important, functional ecosystems are also needed for the provision of key ecosystem services for human well being and for adaptation to the phenomenon of climate change. Various reports prepared by the Intergovernmental Group of Experts on Climate Change (IPCC) of UNEP [33–35] indicate that global warming of the planet is also a factor that accelerates the arrival and spread of infectious diseases. This situation is aggravated by increasing urbanization and the accelerated change in land uses that are taking place around the world, in which natural resources are being overexploited and natural ecosystems are being contaminated. This scenario facilitates the spread of infectious diseases, the consequences of which are accentuated, as is the case of the recently detected link between the high mortality rates from the coronavirus in Madrid and some cities in Northern Italy which have alarming rates of air pollution [56].

Faced with degraded and polluted territories, well structured and functional ecosystems provide society with a large number of environmental goods and services of great value and economic importance, such as clean air and water, carbon storage, pollination, etc. They also play a fundamental role in the fight against climate change, protecting us from epidemics, floods, and other environmental catastrophes.

To respond to these important environmental challenges, policymakers can adopt, on the one hand, engineering or technological strategies; and/or, on the other hand, alternative approaches based on the comprehensively managing natural and social systems in order to increase the benefits that nature provides for human well being, health, and development [46]. GI is defined as a strategically planned network of natural and semi-natural spaces and other environmental elements designed and managed to offer a wide range of ecosystem services [14, 58]. This term is intended to simplify complex ecological concepts related to the functioning of ecosystems and the ecosystem services they provide, making an analogy between the infrastructure of natural systems and the gray infrastructure of human artificial systems, such as road networks or the hydraulic infrastructures themselves. Investing in GI is based on the logic that it will always be more profitable to invest in nature-based solutions than to replace these ecosystem services with human technological solutions [75]. Table 1 shows the main benefits of GI grouped according to main ecosystem service types.

**Table 1:** Potential ecosystem services and benefits of GI

Habitat services	Regulating services
1. Biodiversity/species protection:	1. Climate change adaptation:
a) Habitats for species	a) Mitigating urban heat island effect
b) Permeability for migrating species	b) Strengthening ecosystems' resilience to climate change
c) Connecting habitats	c) Storing floodwater and ameliorating surface water run-off to reduce the risk of flooding
Cultural services	2. Climate change mitigation:
1. Recreation, well being, and health:	a) Carbon sequestration
a) Recreation	b) Encouraging sustainable travel
b) Sense of space and nature	c) Reducing energy use for heating and cooling buildings
c) Cleaner air	d) Providing space for renewable energy
d) Tourism/Ecotourism	Provisioning services
2. Land values:	1. Water management:
a) Positive impact on land and property	a) Sustainable drainage systems, attenuating surface water run-off
3. Culture and communities:	b) Fostering groundwater infiltration
a) Local distinctiveness	c) Removal of pollutants from water
b) Opportunities for education, training and social interactions	2. Food production and security:
c) Tourism opportunities	a) Direct food and fiber production on agricultural land, gardens, and allotments
	b) Keeping potential for agricultural land
	c) Soil development and nutrient cycling
	d) Preventing soil erosion

Source: European Environment Agency (2011)

In the context of Europe, the concept of GI is a fundamental element of the strategies aimed at achieving a climate neutral Europe and protecting natural habitats for the benefit of people, the economy and the planet [14]. This is in line with the formulation of the Green Deal of the European Commission, a new growth strategy based on a green and fair transition which plans to mobilize at least 100,000 million euros during the period 2021–2027 [16]. These types of European policies point to the fact that GI could become a primary strategic factor for European cities and municipalities when facing not only global environmental challenges, but also the economic and social reconstruction that will be necessary after the coronavirus epidemic.

An analysis of GI initiatives in European countries revealed seven major areas where GI approaches have been adopted; namely: ecological networks for biodiversity, connectivity and ecological coherence; multifunctional use of farmland and forests; multifunctional use of coastal areas; freshwater and wetlands management and restoration; urban GI; gray infrastructure mitigation; and GI mapping for planning [50]. The present work specifically addresses this last initiative, GI mapping for planning, and its main contribution is to offer a methodology that identifies and evaluates GI at the municipal level, taking the region of Andalusia (Spain) as a reference. Based on this methodology, the following objectives are pursued in this work. First, to identify and characterize the elements of GI at the municipal level, both in urban and rural settings, through the use of GIS technology. Secondly, to analyze the possible clusters that group the municipalities of Andalusia based not only on the state of their GI but also based on the socioeconomic indicators of the municipalities where the GI is located. And, finally, in light of the the results obtained, propose approaches to public management aimed at prioritizing the ecosystem services of the GI and addressing possible demographic and/or socioeconomic problems in the municipalities.

## Methodology

GIS (geographic information system) technology is widely used in environmental studies. Along these lines, Rüdisser et al. [70] analyze the parameters of the linear regression model together with exhaustive spatial data from GIS to spatially predict the values of the soil biological quality index. Wang et al. [79] investigate the solar, wind, biomass, geothermal, and hydroelectric potential within Fukushima Prefecture (Japan). Zolin et al. [81], focus on the spatialization and production of different information plans. Xiao et al. [80] estimate and map the biological diversity and ecosystem services in the municipality of Chongqing (China). Nagy et al. [54] combine the analysis of quantitative data with a computer mapping technique. More recently, several works have been published that relate GIS technology with environmental indicators [2, 52, 71].

Our study commenced with a selection of indicators related to GI and the socioeconomic sustainability of the municipalities of Andalusia. Studies related to the establishment of GI indicators in the international arena have been reviewed in order to minimize the subjectivity associated with the methodological process for selecting indicators and establishing assessment thresholds for each one [1, 15, 60, 69]. Various reports from national and international institutions have also been consulted, establishing thresholds for some of the indicators used European Commission 2016 [10, 22, 55]. Finally, consultations have been carried out with a panel of experts, from both the academic field and from public management, the Ministry of Agriculture, Livestock, Fisheries and Sustainable Development, the Andalusian Environment and Water Agency as well as technicians and heads of City Councils.

The final set of indicators has been selected by applying representativeness and availability criteria in the following regional and national statistical and cartographic information sources: Environmental Information Network of Andalusia (REDIAM), the Andalusian Multiterritorial Statistical Information System (SIMA), Atlas of Andalusia, National Statistics Institute (INE), and Spatial Data Infrastructure of Spain (IDEE).

**Fig. 2:**
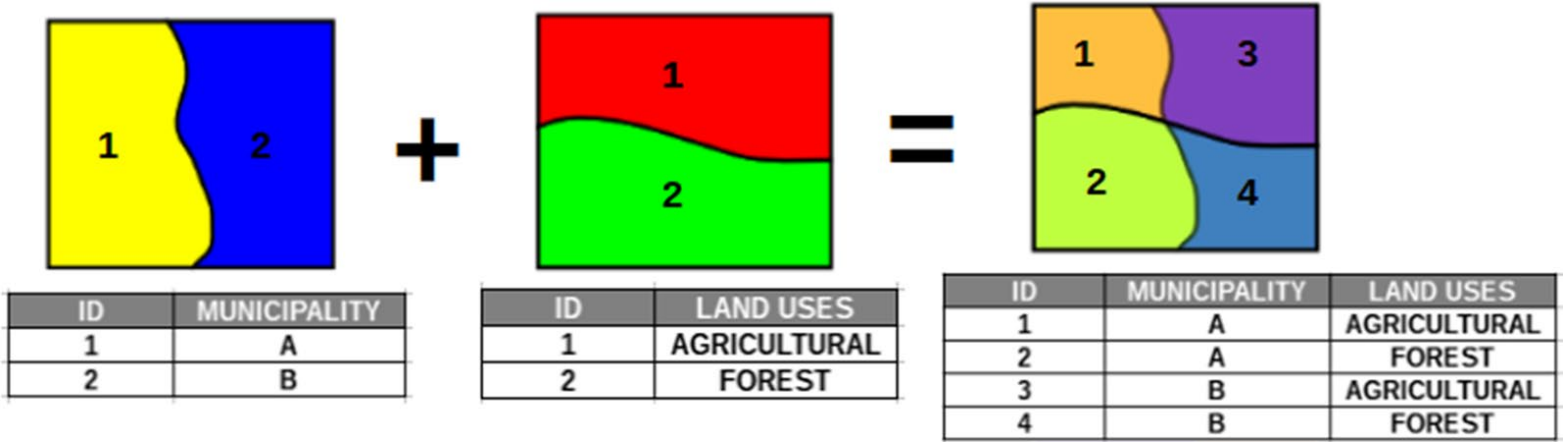
Example of Intersection between two layers of polygons (geometries and table of attributes). Source: Own Compilation

Once the statistical information and cartography have been selected, spatial analysis operations have been carried out through GIS technology (ArcGis 10.4.1) that have allowed the GI to be scaled in each of the municipalities of Andalusia, in addition to applying a socioeconomic characterization of each of them. The geoprocessing method used is the intersection method, in which the input elements are cut from another layer superimposed on the first. The result is a new layer that collects the spatial combination of the different elements that make up both layers (Fig. 2).

The joint geoprocessing of the different types of geographic databases selected through GIS technology has allowed us to establish a novel system of useful indicators to delimit and characterize GI at the municipal level, both from an environmental and socioeconomic point of view.

In order to contrast the validity of the results obtained in the selection of the indicators through the implementation of GIS, a cluster analysis was carried out using the indicators in Tables 2 and 3. This is a method that allows individuals within a specific municipality to be grouped based on a series of variables, whose versatility is made manifest in the variety of areas in which it has been used, such as medicine [6, 53], economics [3, 64] or even local development [9, 51],among others. This methodology presents several types of clusters, such as the hierarchical cluster, in which the division by groups follows the shape of a tree [40], spatial clustering, based on the density of noise applications [30], or the K-means method, which is used in a multitude of research areas [47]. This latter clustering method has been chosen due to the nature of the data and its availability [49].

**Table 2:** GI indicators

	Indicator	Variable	Criteria	Type	Source	Year
Indentification indicators	Core area	Protected Natural Area	> 50% of Municipal area is a Natura 2000 site	Vector Polygon	REDIAM	2018
		Habitats of Community Interest	> 50% of Municipal area is considered a Habitat of Community Interest	Vector Polygon	REDIAM	2018
	Buffer zones /ecological corridors	Buffer zones/ecological corridors	> 50% of Municipal area is considered an Important Area for Ecological Connectivity	Vector Polygon	REDIAM	2013
Characterization indicators	Biodiversity	Areas of rich biodiversity	> 10% of Municipal Area is in the Biodiversity Atlas	Vector Polygon	ATLAS OF ANDALUSIA	2005
	Fragmentation	Artificial surface	> 10% of Municipal Area is artificial surface	Table	SIMA	2017
			> 10% of Municipal Area is Industrial Agriculture under plastic	Table	*SIMA*	2019

Source: Own Compilation

**Table 3:** Socioeconomic sustainability indicators

	Indicator	Criteria	Type	Source	Year
Demographic indicators	Evolution in population 1996–2019	Variation in population ( ±) during this period	Table	INE	2019
	Municipalities with small populations	Municipalities with population of < 1000 in 2019	Table	INE	2019
		Municipalities with population of > 1000 in 1996	Table	INE	2019
	Population density	Municipalities with population density < 12.5 inhabitants /km^2^	Table	SIMA	2019
		Municipalities with population density > 500 inhabitants /km^2^	Table	SIMA	2017
Economic indicators	Annual declared income	Municipalities with average declared income < regional average	Table	SIMA	2017
		Municipalities with average declared income > regional average	Table	SIMA	2017
		Municipalities with average incomes well below regional average (< €7000 p.a.)	Table	SIMA	2017
		Municipalities above average income (> €18,000 p.a.)	Table	SIMA	2017
	Municipal rate of unemployment	Municipalities with unemployment rates < regional average	Table	SIMA	2019
		Municipalities with unemployment rates > regional average	Table	SIMA	2019

Source: Own compilation

The procedure follows a simple process. First, the user decides the number of clusters into which they want to divide the sample. Each element of the data sample that is assigned to a centroid is considered to be a cluster. The centroid of each cluster is updated based on the objects assigned to the cluster. The allocation and update steps are repeated until no data point modifies the groups or until the centroids remain the same [43]. Specifically, eleven indicators have been used to carry out this analysis: five related to GI and six related to the socioeconomic sustainability of the municipalities of Andalusia.

### GI Indicators

The indicators related to GI are divided into two categories: identification and characterization indicators Table 2.

#### Indicators for the identification of the GI

**Fig. 3:**
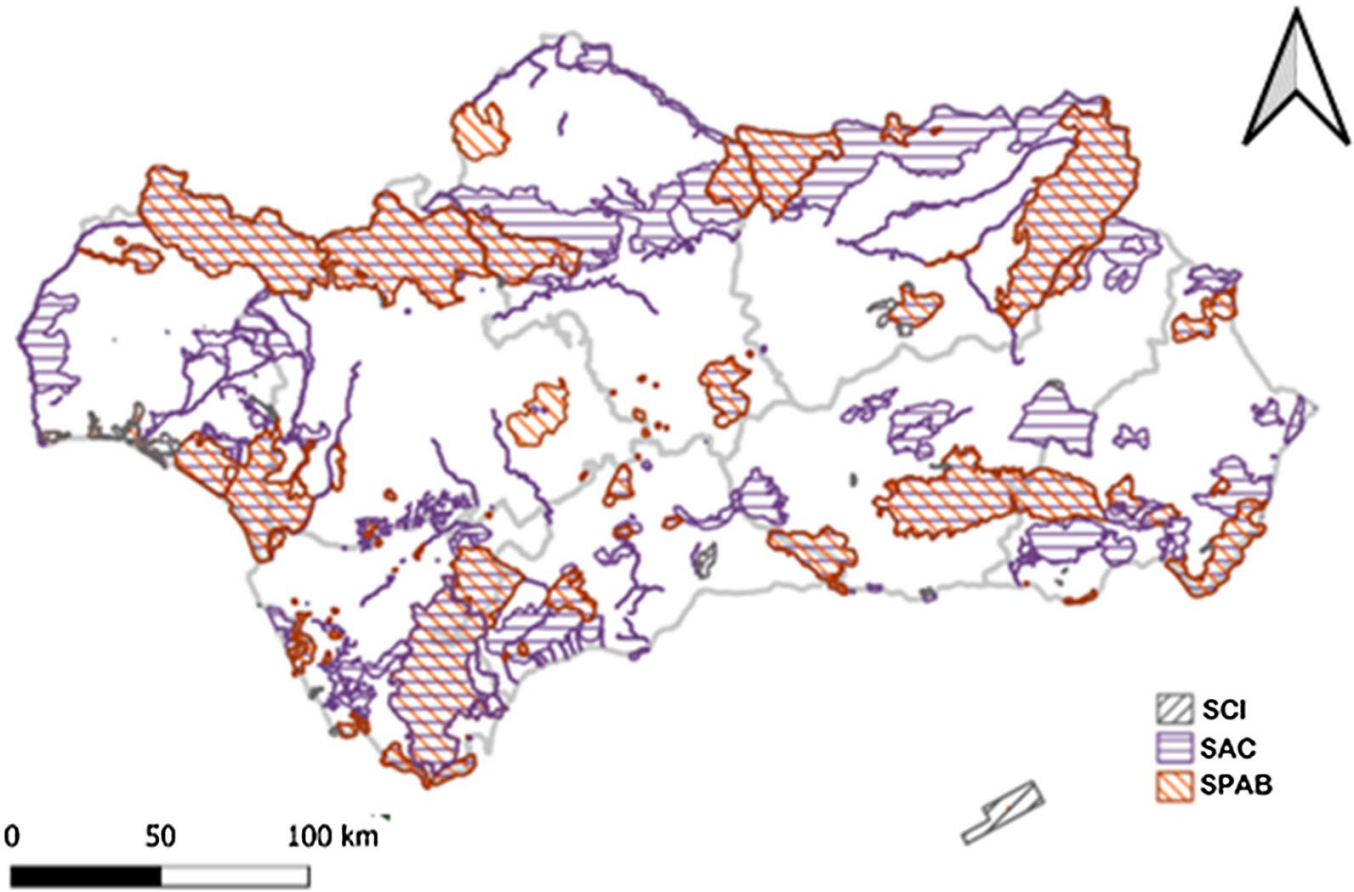
Network Natural Protected Areas of Andalusia (Natura 2000). *SCI: Site of community importance, SAC: Special area of conservation, SPAB: Special protected areas under Bird Directive.* Source: [67]

For a proper identification of the GI in the municipalities of Andalusia, the classification guide of the European Commission [13] has been used, which differentiates:

*Core Areas *These are areas where conservation is a priority, even if that area is not legally protected. These areas have included not only all the areas located in Protected Natural Spaces of Andalusia, but also those well-preserved ecosystems and areas of high ecological value [75]. For the identification of these core areas, in our work we have used the information layer of the Network of Protected Natural Spaces of Andalusia; and to delimit well-preserved ecosystems, the Habitats of Community Interest layer of the European Directive 92/43/EEC has been used (Fig. 3). Both sources of information are available on REDIAM.

This study has used the, *the Core Area Indicator to identify those municipalities that have more than 50% of their territory included in the network of Protected Natural Areas of the European Union (established by Directive 92/43*/*EEC and known as Natura 2000) and*/*or as Habitats of Community Interest.*

*Buffer zones and/or ecological corridors* One of the main purposes of GI is to guarantee the ecological connectivity of a territory, since this is essential to maintain the ecological flows of energy and materials and, more particularly, natural heritage and biodiversity [65, 68, 78].

For the identification of important areas for ecological connectivity has been used the mapping of the Master Plan for the Improvement of Ecological Connectivity in Andalusia, developed by the regional government of Andalusia [39].

By means of the *Indicator for buffer zones/ecological corridors, those municipalities with more than 50% of their territory are included in areas that are important for ecological connectivity, applying the criteria as established in the Master Plan for the improvement of the Ecological Connectivity of Andalusia. Landscapes of interest for connectivity (LIC) and Reinforcement Areas (RA).*

#### Indicators for the characterization of GI

In order to assess the conservation status and integrity of GI in the Andalusian municipalities, based on information available at the municipal level (REDIAM, SIMA, Atlas de Andalucía, and IDEE), three criteria have been selected: biodiversity, fragmentation and state of conservation of aquifers.

**Fig. 4:**
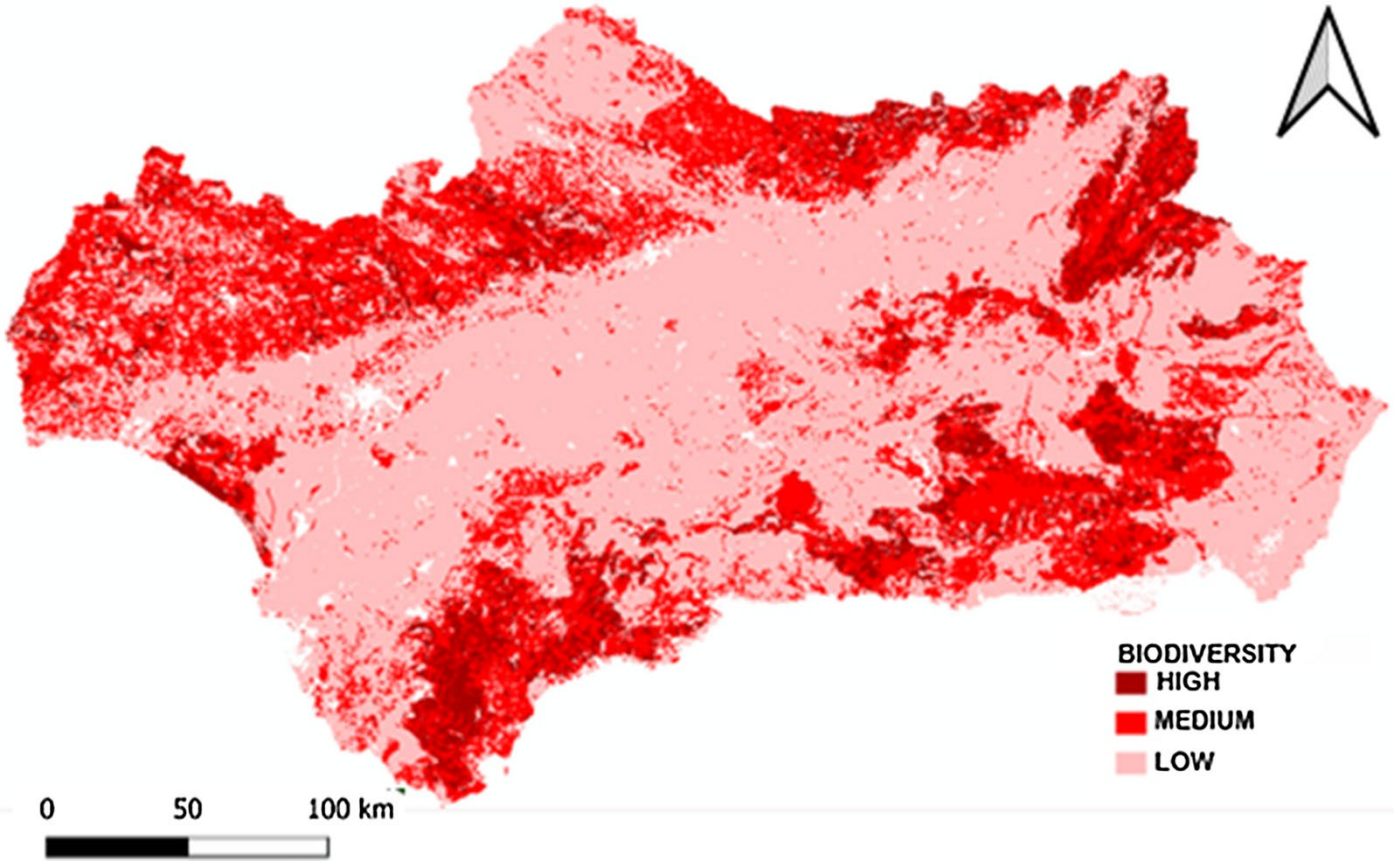
Biodiversity Map of Andalusia. Source: Atlas of Andalusia and REDIAM (2005)

*Biodiversity* For the elaboration of the biodiversity indicator, it has been used the biodiversity map included in the Atlas of Andalusia, volume II, which reflects the variation and relative abundance of habitats and species in the region has been used to develop a GI biodiversity indicator for this region [66]. This biodiversity map was prepared from a series of normalized and standardized variables in the context of REDIAM. Some of the variables of note used for its development are the typology and distribution of plant associations, the different uses of the soil, the distribution and endemicity of the main taxa of flora and fauna and the degree of threat of natural and semi-natural habitats. From this information, the level of plant diversity is represented and natural ecosystems are classified (high, medium, and low), and in cultivated areas, structural diversity is also classified (high, medium, and low) (Fig. 4).

An indicator has been developed to select those municipalities that contribute most to the conservation of biodiversity. Specifically, *the Biodiversity Indicator has been selected to represent all those municipalities in which more than 10% of their territory in classified High Biodiversity areas according to the Atlas of Andalusia (2005).*

*Fragmentation *To assess the degree of fragmentation of the region's GI, two indicators were used, based on the information available from SIMA. First, the percentage of artificial soil of each of the Andalusian municipalities in 2017 has been taken into consideration, according to the criteria established by Eurostat [19] and, secondly, the area occupied by intensive agriculture under plastic has been considered for the year 2018.

*The Fragmentation Indicator used in this work includes those municipalities that have more than 10% of their territory occupied by artificial surfaces or industrial agriculture under plastic.*

### Socioeconomic sustainability indicators.

In relation to socioeconomic sustainability indicators, it must be pointed out that the GI of Andalusia has its origin in a unique geology and climate, although its structure and operation is highly conditioned by the human activities that have taken place over millennia in the region [37, 38, 74].

#### Demographic indicators

The following indicators have been used to analyze the population variable in the study:

*Changes in population in the period 1996–2019 *Measured as a percentage of variation between the two periods.

*Municipalities with a small population *Measured as a percentage of the total of municipalities with less than 1,000 inhabitants in both 1996 and 2019.

*Population density *Measured as the number of municipalities with a population density of less than 12.5 inhabitants/km^2^ (municipalities at risk of depopulation) or greater than 500 inhabitants/km^2^ (municipalities at risk of overcrowding).

The reason for including these three indicators is to not only understand population levels (represented by the population density of each municipality) but also the depopulation trends of recent years (represented both by the proportion of municipalities with fewer than 1000 inhabitants and the evolution of the population between 1996 and 2019).

#### Economic indicators

The economic status of the municipalities of Andalusia has been measured using the following indicators:

*Average declared income* Represented by the number of municipalities with higher and lower declared income than the regional average, as well as the number of municipalities with average incomes of less than € 7,000 of per annum and municipalities with average incomes of more than € 18,000 per annum.

*Municipal unemployment rate *Measured as the number of municipalities with an unemployment rate higher or lower than the regional average.

Using these two indicators allows us to gain a broader perspective of the economy both in terms of income and employment Table 3.

## Results

After using the selected indicators, the results obtained are presented below. Firstly, a classification of the municipalities of Andalusia has been developed in relation to the conservation status of their GIs, these have subsequently been linked to the socioeconomic situation of each of these localities in order to identify possible trends and general processes regarding environmental management, land use, and demographic and economic evolution in the region.

### GI, biodiversity and fragmentation

The cartographic representation of the location indicators shows an extensive and well-configured GI network in Andalusia in the mountainous areas of the region (Sierra Morena and the Subbética and Penibética mountain ranges) and more fragmented network in the coastal strip and the Guadalquivir valley.

On the other hand, the GI buffer zones encompass a series of diverse landscapes ranging from low and medium mountain areas to extensive patchwork of agricultural crops, which stand out for their natural value and their ability to adapt to ecological flows.

**Fig. 5:**
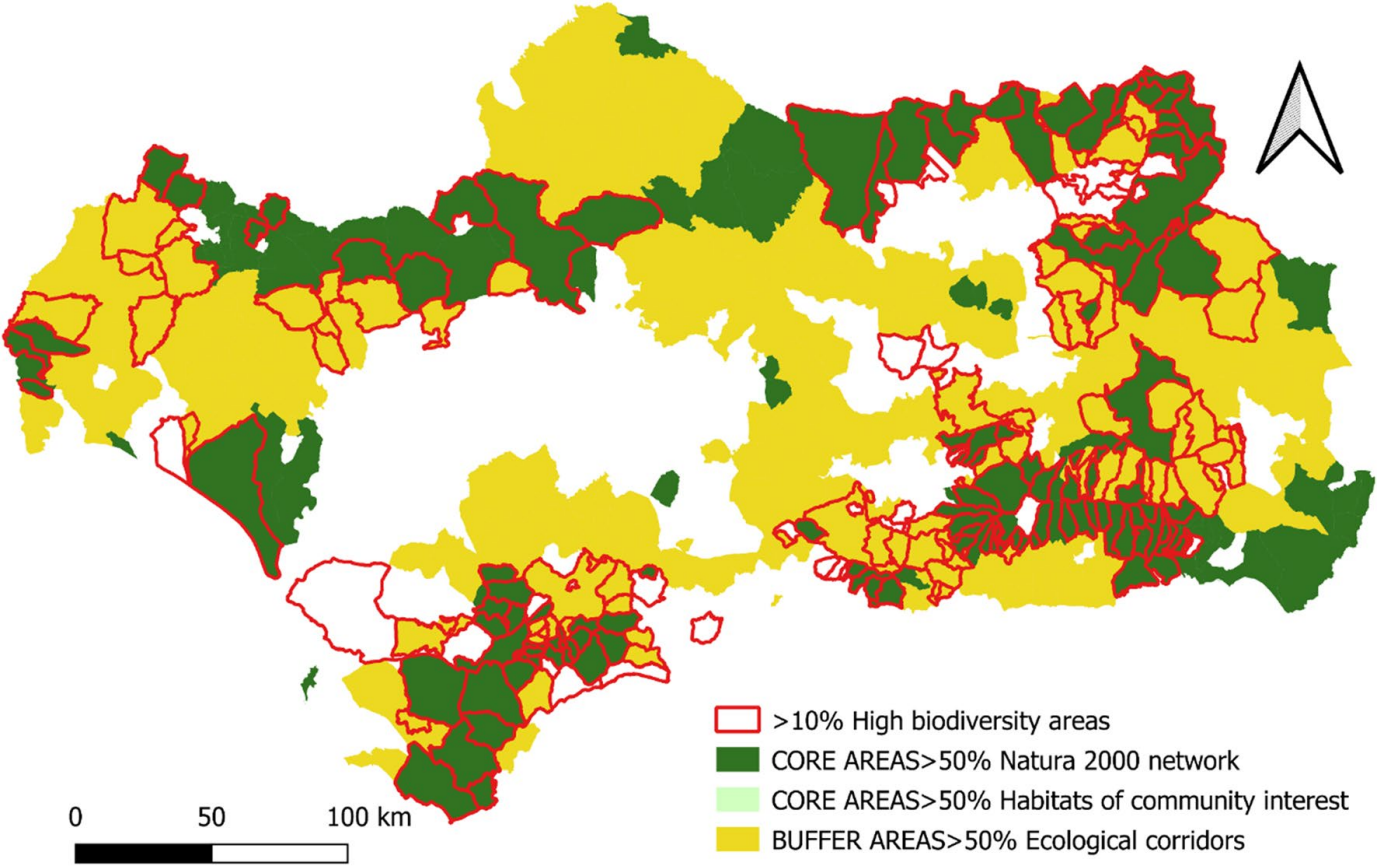
GI and municipalities with high biodiversity. Source: Own Compilation

The Core Areas, in their entirety, are attached to the network of Protected Natural Spaces and specifically to a large number of municipalities that include Habitats of Community Interest. Buffer Zones, in contrast, are widely distributed throughout the region and act as ecological corridors, fulfilling the ecological connectivity function of the Core Areas (Fig. 5).

The inland Core Areas are made up mostly of forest and high mountain ecosystems, most of which are public property, in which there is rich and unique biological diversity as a result of variable environmental conditions and minimal traditional ecological human management.

**Fig. 6:**
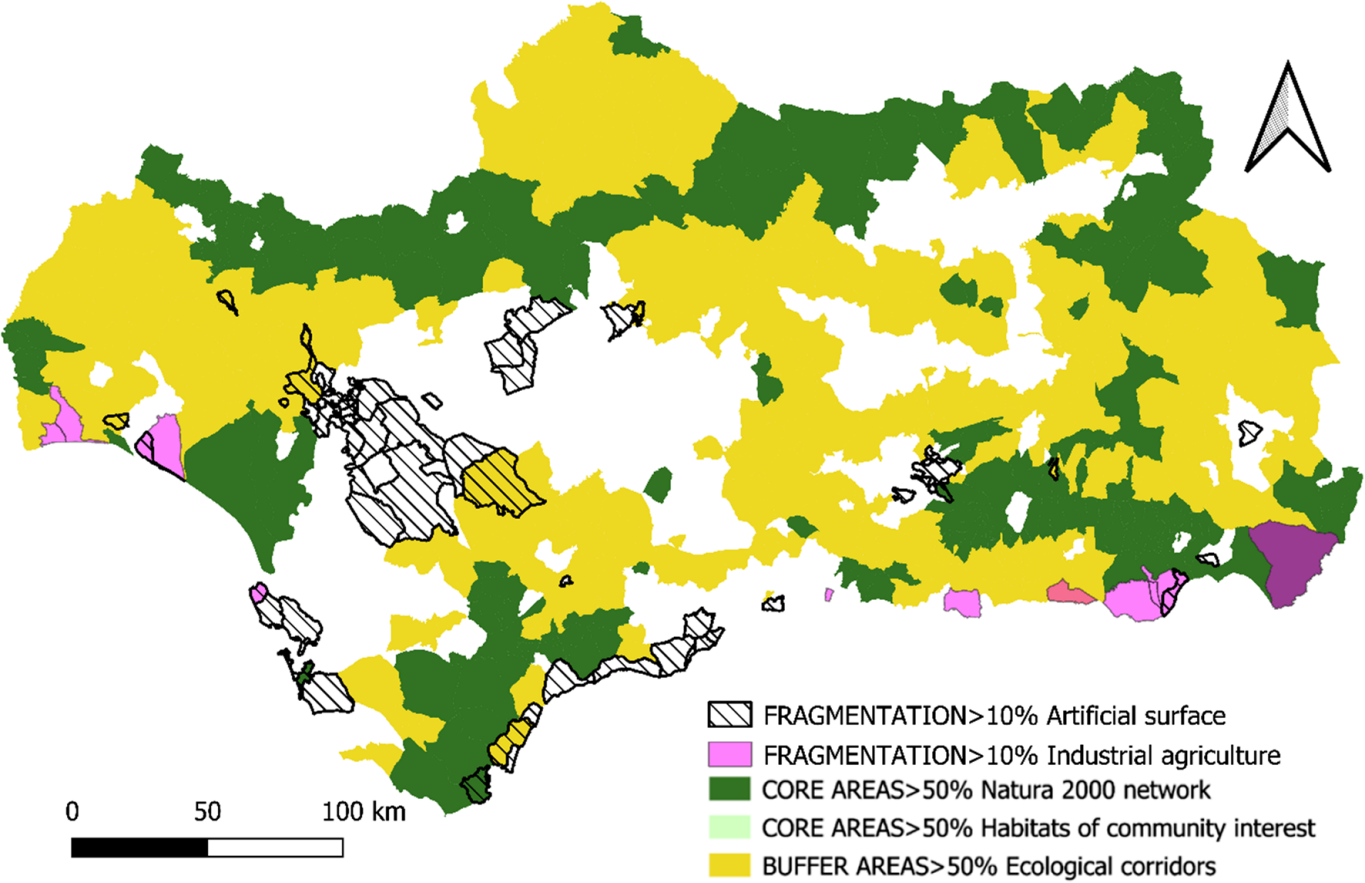
Fragmentation. Source: Own Compilation

The Core Areas of the coastal and semi-arid spaces in the extreme southeast of the region are characterized by ecosystems of great uniqueness and rarity in the context of the European Union, but of a smaller area, more fragmented and with less biodiversity compared to the ecosystems of the inland Core Areas (Fig. 6).

In this regard, as shown in Fig. 6, many of the Andalusian coastal municipalities have more than 10% of their surface occupied by urbanized and artificial soil (mainly the Costa de Sol and Cádiz) and by intensive agriculture under plastic (coast of Almería, western Granada and the coast of Huelva). In some coastal municipalities, more than 60% of their first coastal kilometer is urbanized: Torremolinos (73.8%), Fuengirola (73.4%), Malaga (72.3%), Benalmádena (69.3%), Mijas (61.7%). There are also cases where more than 50% of the municipal surface is occupied by greenhouses, namely El Ejido (56.94%) and La Mojonera (60%).

In addition to the fragmentation of the coastline, there are large pockets of artificial soil around the urban peripheries of some provincial capitals of the region (Seville, Granada, Malaga, and Almería).

### GI and demography

**Fig. 7:**
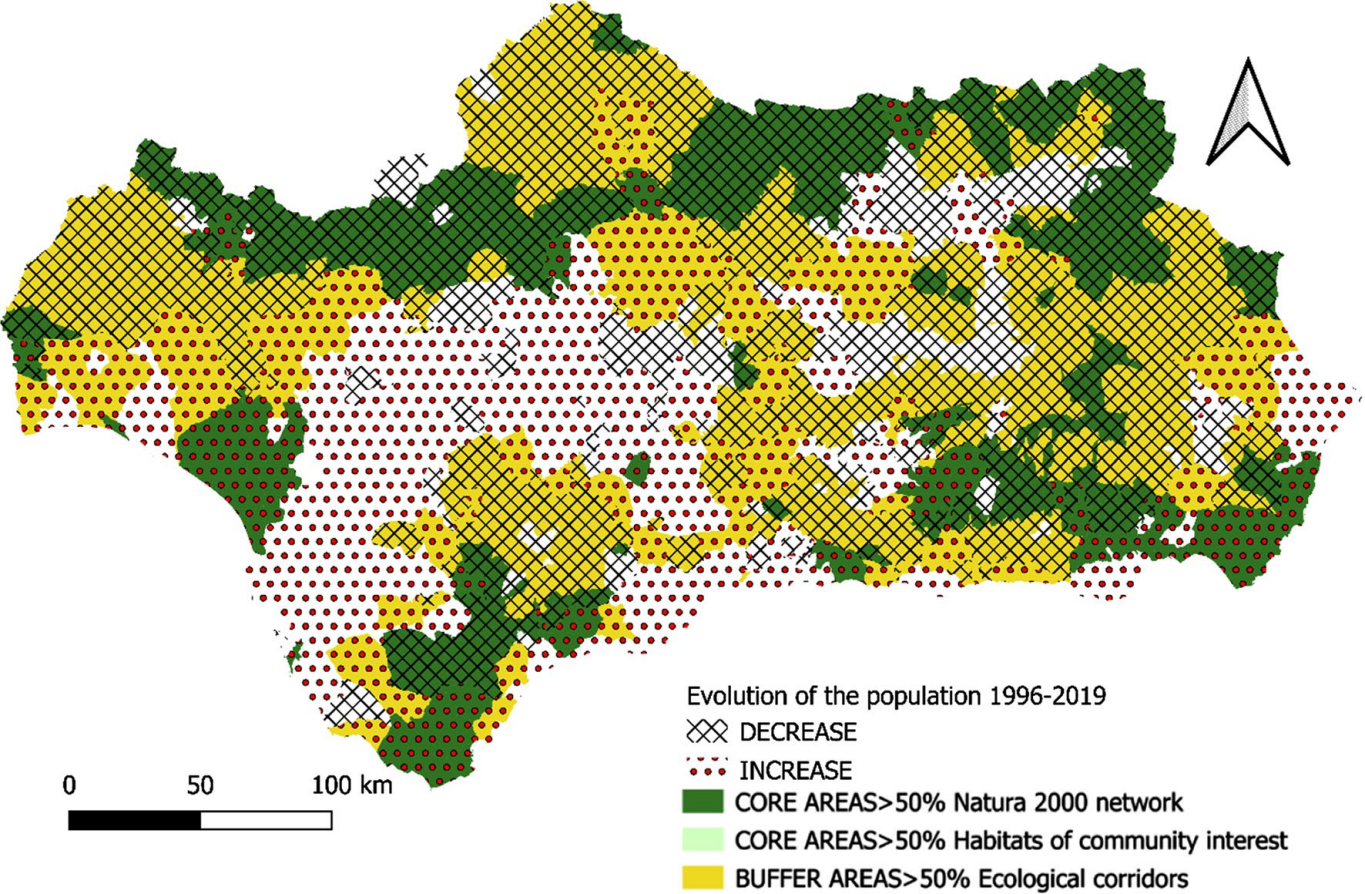
Evolution of the population between 1996 and 2019. Source: Own Compilation

Since the mid-twentieth century, rural Andalusian municipalities have suffered continuous demographic losses as a result of the heavy emigration of the population to the coast and the large metropolitan areas of the region (Fig. 7).

The municipalities in the Core Areas of the GI are not oblivious to this general dynamic since the hinterland and most peripheral areas lose population while the coasts and areas closest to large metropolitan areas gain population. However, there are some inland municipalities that evince a trend away from this general dynamic of population regression during the period 1996–2019 (Guejar Sierra, Monachil, Huétor-Santillán, Aracena, El Bosque, Paterna del Rio, and Fondón). The common characteristic of all these municipalities is their inclusion in the Andalusian Network of Natural Parks and the weight of rural tourism in their local economy.

**Fig. 8:**
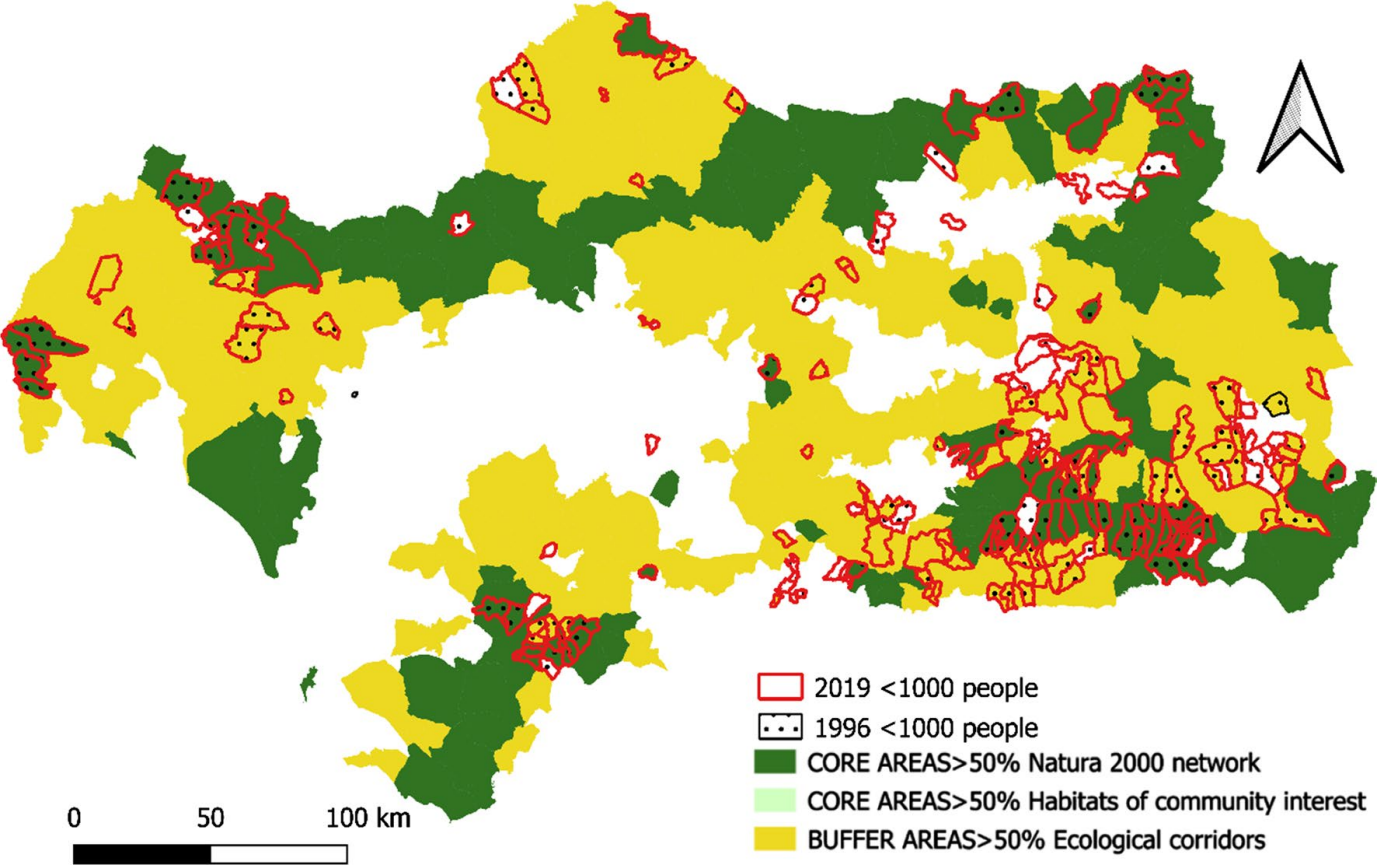
Municipalities with population < 1000 inhabitants in 2019 and in 1996. Source: Own Compilation

Regarding the indicator referring to municipalities with a population of fewer than 1,000 inhabitants during the period 1996–2019, there is no difference in dynamics observed between municipalities located inside and outside the GI areas (Fig. 8). In general, the number of municipalities in the region with fewer than 1,000 inhabitants have remained stable during this period.

**Fig. 9:**
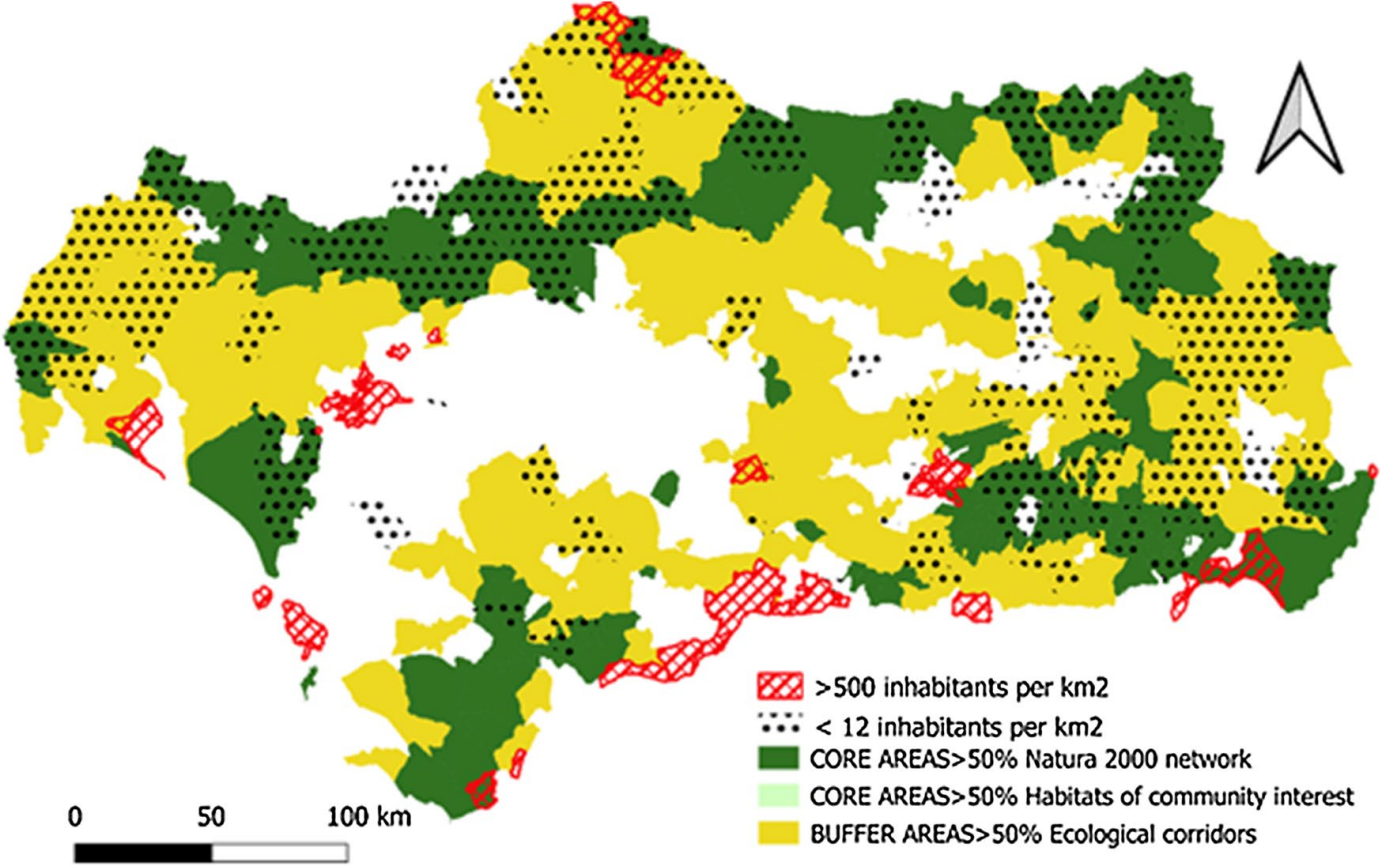
Municipalities with low and high population density. Source: Own Compilation

With regards to the population density of the municipalities of Andalusia, the previously mentioned phenomenon of migration to coastal areas is very obvious (Fig. 9). Almost all of the municipalities with serious risk of depopulation (< 12 inhabitants/km^2^) are located further inland from the GI Core Areas and Buffer Zones. Those areas with high population density (> 500 inhabitants/km^2^) are located, for the most part, outside the GI Core Areas, in coastal areas and, occasionally, in the metropolitan areas of the provincial capitals.

### GI and socioeconomic indicators

**Fig. 10:**
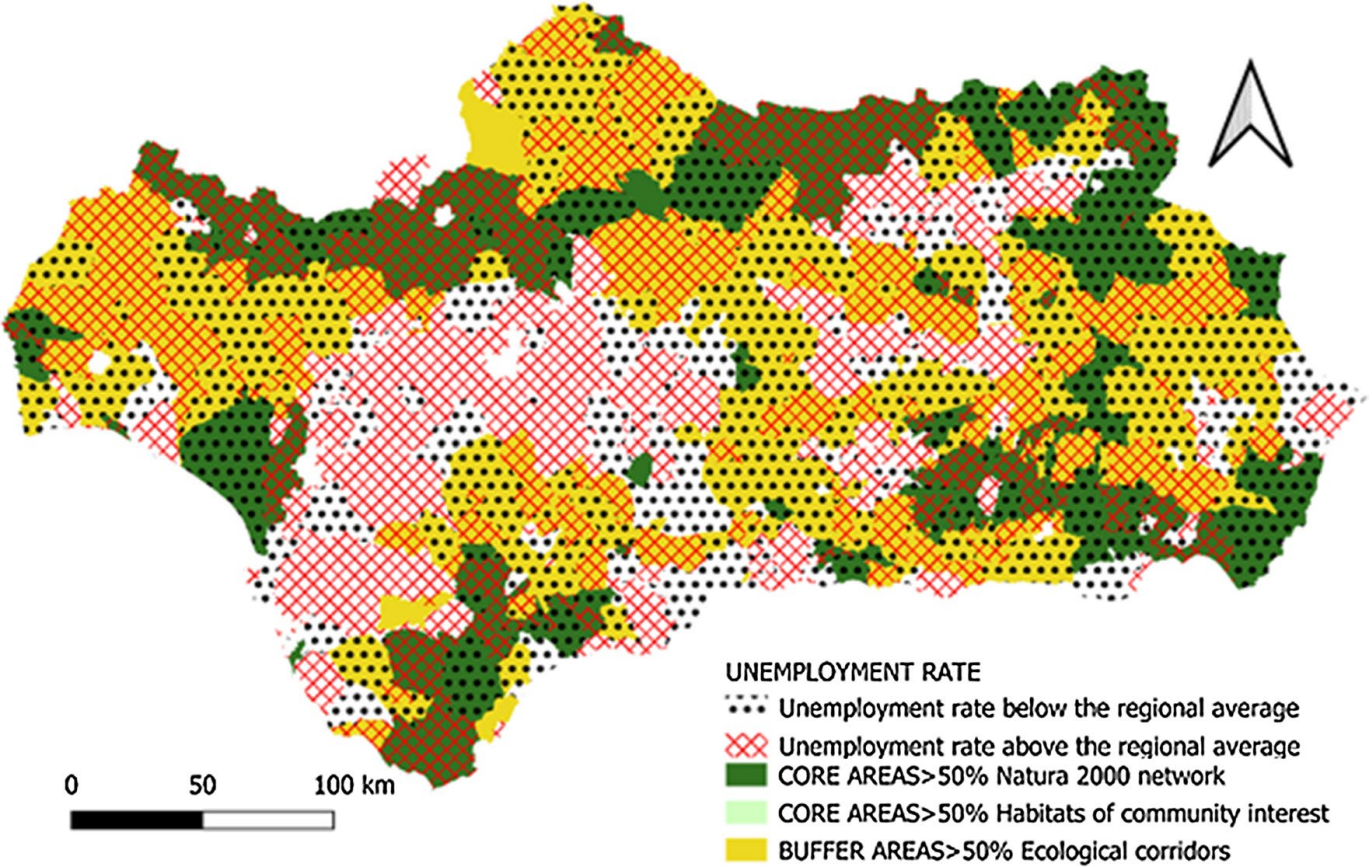
Unemployment rate. Source: Own Compilation

Many of the municipalities with unemployment rates above the regional average are located in coastal and urban areas (Cuevas del Almanzora, Almería, Roquetas de Mar, Almuñecar, Mijas, Marbella Tarifa, Conil de la Frontera, Cádiz, Punta Umbría, Sevilla, Córdoba, etc.). This can be explained by the strong impact caused by the financial crisis on the construction sector and the bursting of the housing bubble (Fig. 10). As a counterpoint to the above, it is interesting to note that a good many of these urban municipalities, whose employment has hitherto been less affected by the financial crisis, have an agrarian-based economy (Níjar, El Ejido, Adra, Almonte), although in some of these, tourism also plays an important role in the economy (Mójacar, Carboneras, Nerja, Málaga). Thus, it becomes clear that these two sectors of activity, agriculture and tourism, are helping to boost the employment rate in Andalusia.

Part of the results and trends obtained in this section regarding the unemployment rate are similar to those obtained in previous studies [29].

**Fig. 11:**
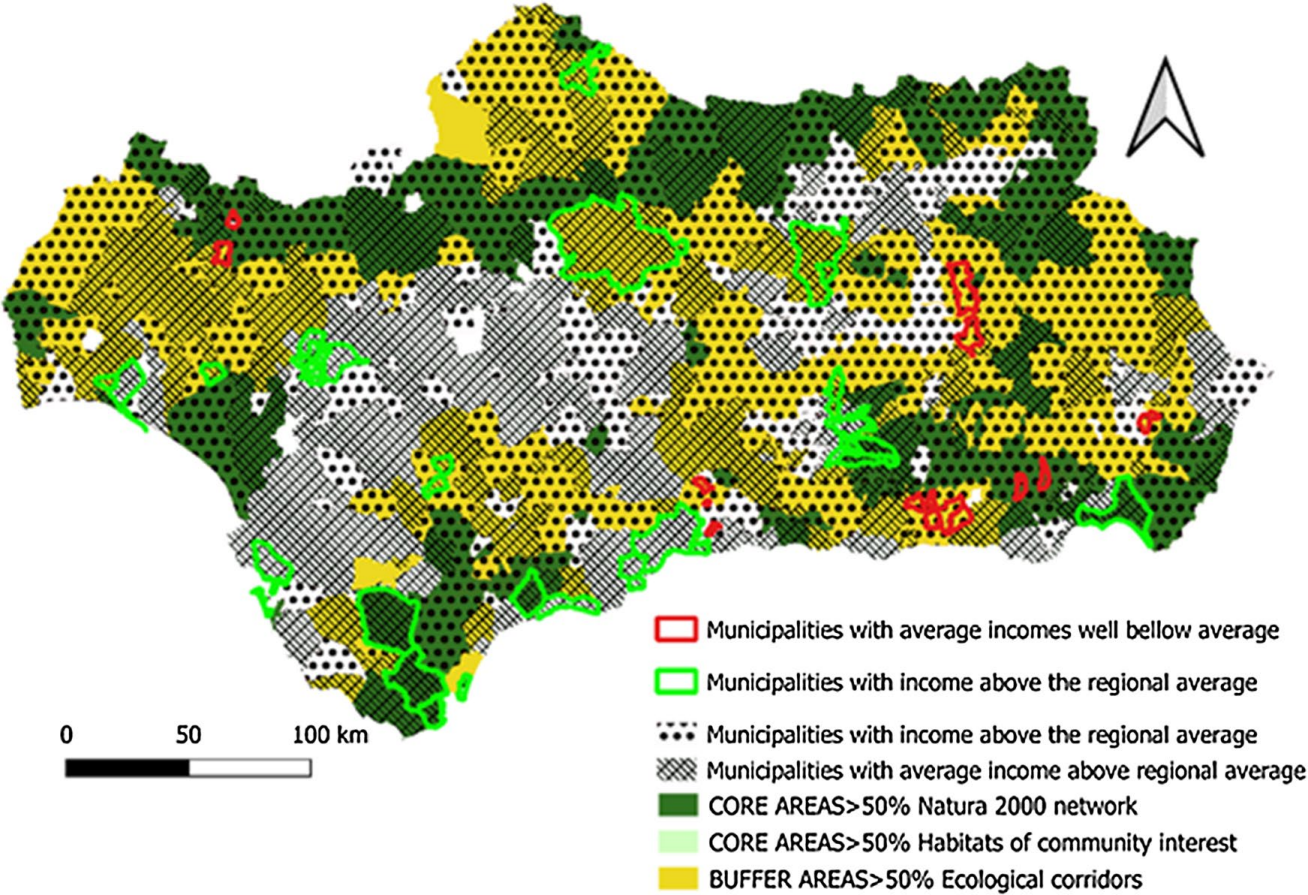
Per capita income distribution. Source: Own Compilation

Regarding per capita income (Fig. 11), as with demographic indicators, those municipalities with incomes above the regional average are located principally in coastal towns and around the large metropolitan areas. These are the areas where the largest companies in the region are located and there is greater economic dynamism. A positive aspect to highlight is the good rate of employment and income data offered by some rural populations located in GI areas and within the scope of the network of Protected Natural Areas in the region. A good number of municipalities in the natural parks of Sierra María-Los Vélez, Sierra de Cazorla, Segura and Las Villas, Sierra Norte, Aracena and Picos de Aroche, Alcornocales, Sierra de las Nieves, Sierra Nevada, or Sierra de Baza show more positive data as compared to the regional average, both in terms of unemployment rates and income.

### Cluster analysis of indicators used

**Fig. 12:**
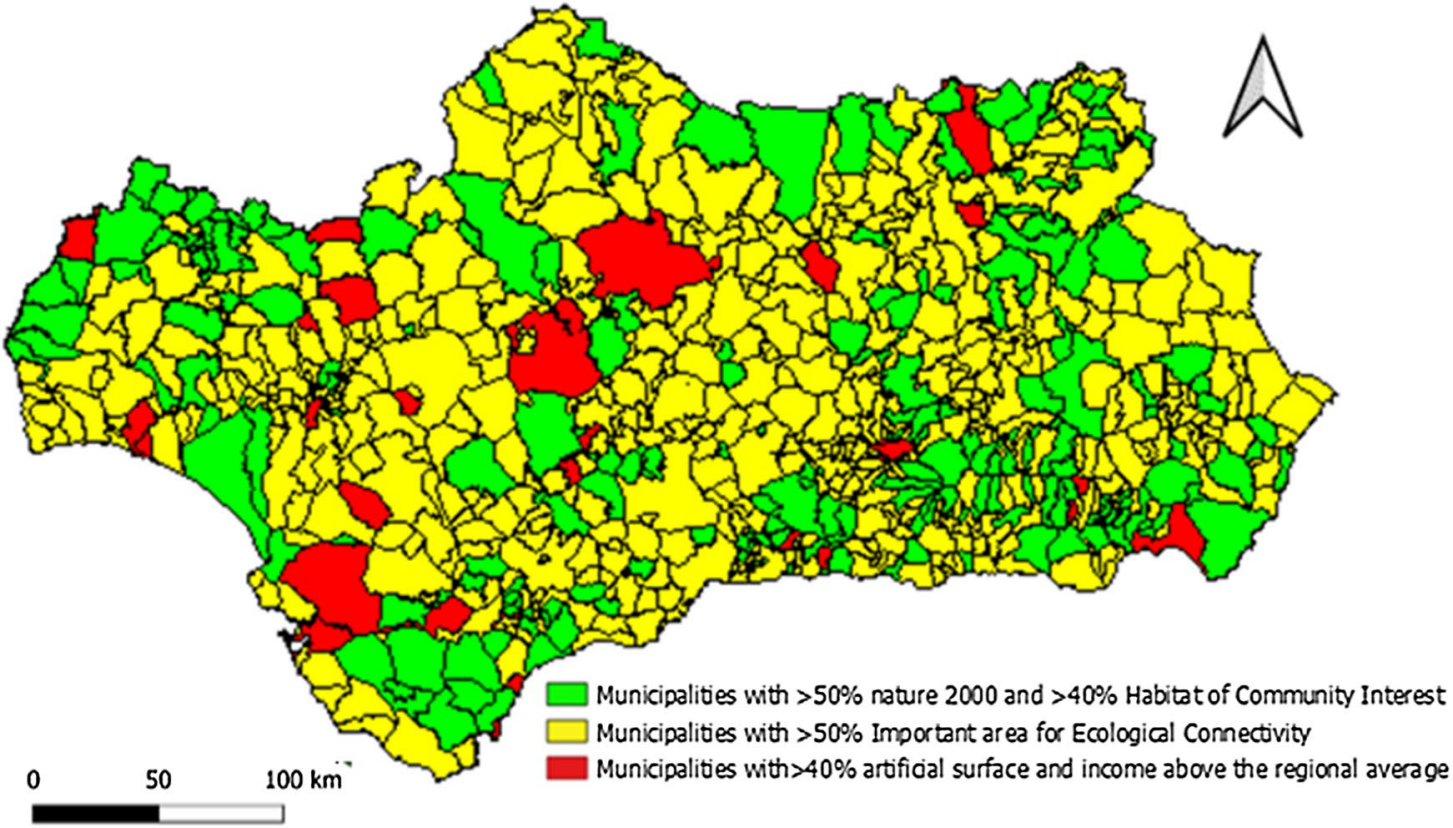
Cluster of the municipalities of Almería Source: Own Compilation

By means of a cluster analysis of the indicators used, a comparison will be made to those results obtained through GIS technology. The indicator data have been standardized and the R NbClust package has been used to decide the relevant number of groups based on various indicators [7]. In this instance, it was deemed that three was the ideal number of groups. Next, a clustering of Andalusian municipalities has been carried out using the K-means method. The results are reflected in Fig. 12.

The results obtained through the cluster analysis of the indicators offer a similar trend to those obtained through the analysis by means of geographical information systems.

The territories of the municipalities in inland and mountainous areas (green areas) are those which most closely configure the Core Areas of the IGs in Andalusia, as they have the largest surface area of Natural Areas, Habitats of Community Interest and areas of high-quality biodiversity.

On the other hand, if we exclude certain coastal areas and large metropolitan areas (red areas) where the fragmentation of ecosystems is greater, the cluster analysis confirms once again the adequate ecological connectivity between the IGs of the region (yellow areas).

From the socioeconomic point of view, it also reconfirms the trend that municipalities with lower population, higher unemployment and lower socioeconomic level are more concentrated in the core areas and zones of ecological connectivity of the IGs (green and yellow areas), while those with higher population and socioeconomic level are located in the coastal areas and metropolitan areas (red areas).

## Discussion

The crisis caused by the global pandemic of COVID-19 has revealed how fragile and vulnerable the human species is and how interconnected the planet is to global phenomena, both environmental and social. There is scientific evidence that biodiversity has positive impact on the productivity of ecosystems and the ecosystem services they provide, adapting to the adverse effects of climate change or offering protection against infectious diseases or pandemics [20, 24, 42, 44, 45, 48].

Likewise, it has also been shown that, in the face of degraded and polluted areas, the well-preserved ecosystems with GI provide society with a protection barrier against pathogens and infections. In particular, these ecosystems are a source of environmental goods and services of great value and economic importance, such as clean water and air, carbon storage or protection against the effects of climate change [72, 73, 77]. This is recognized by the European Union, in the recent 2030 Biodiversity Strategy, where it is pointed out that the risk of the appearance and spread of infectious diseases increases as nature or the proper functioning of ecosystems is destroyed. This strategy identifies that investments in the protection and restoration of nature will be essential for the recovery of the European economy after the COVID-19 crisis, as well as that it will be crucial to avoid falling back into old harmful habits [17, 18]. According to the aforementioned strategy, the European Green Deal "*the EU's growth strategy will be the compass for our recovery, ensuring that the economy serves people and society and gives back to nature more than it takes away*".

Given this future scenario in which Europe is considering a green economic reconstruction, and based on the results obtained in this work, it is considered a priority that the different European regions carry out an adequate assessment and diagnosis of their different GIs as these are responsible for generating key ecosystem services for the quality of life and human well being.

Currently, almost 32.25% of the land area of Andalusia is protected. With 2,825,347.20 ha, this region comprises 21.19% of the entire Natura 2000 sites in Spain (Junta de Andalucía 2017). Andalusia possesses significantly above average number of species compared to other countries of Atlantic Europe and many Mediterranean countries, reaching 56% of the taxa of Community Interest in the Mediterranean region in its territory [36]. These figures are an indication of the key role that this network plays in the configuration of Andalusian GIs and, therefore, in the provision of ecosystem services to the region's population. However, as it is a very heterogeneous region in terms of environments and land use, this biodiversity is not distributed evenly throughout the territory.

The historical absence of market valuation of the ecosystem services provided by the GI in many regions of the planet has led to their overexploitation or deterioration as a consequence of the abandonment of practices compatible with their conservation. This is the case in Andalusia, (see Figs. 5, 6, 7, 8, 9, 10, 11, 12) where the area originally occupied by GI in the coastal strip and in large cities has decreased considerably in recent decades as a consequence of population growth, urbanization and the development of intensive agriculture. Furthermore, these phenomena have caused the fragmentation, decrease, and deterioration of the existing GI [12, 28, 37, 76].

In this sense, the work carried out within the framework of the United Nations Millennium Ecosystem Assessment Initiative (2012) determined that 77% of the evaluated Andalusian coastal ecosystem services were being degraded or being used unsustainably [8].

In contrast, the best preserved GIs have been identified in the rural inland areas of this region, today subjected to the phenomenon of depopulation (Figs. 5, 7, and 9). These infrastructures are responsible for providing, directly or indirectly, key ecosystem services for the quality of life of the entire Andalusian population, including those of the major cities in the region.

**Fig. 13:**
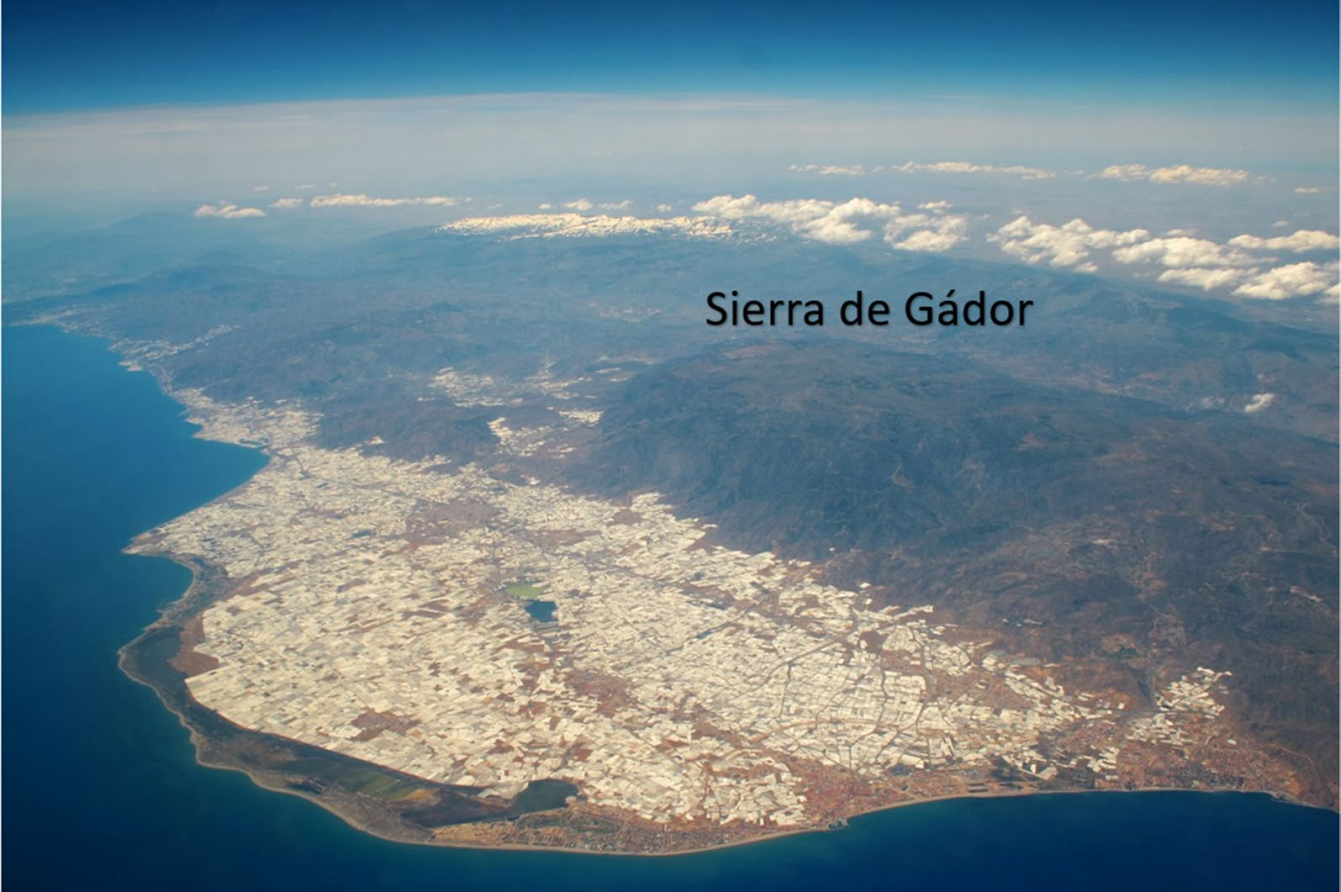
Intensive agriculture in the Poniente Almeriense and Sierra de Gádor regions. Source: Almería Free Tours. https://www.raizes.es/en/postexperiencia/espanol-almeria-freetour/

Perhaps the most paradigmatic case of this contribution is the Sierra de Gádor (Fig. 13), a mountainous massif located in the province of Almería, in charge of supplying the necessary water resources for one of the most productive agricultural zones in Europe and for supplying half of the population of the province of Almería,that is, the inhabitants of the capital Almería, El Poniente and Berja [4, 11, 59].

## Conclusions

In order to address the lack of identification and valuation of the ecosystem services provided by GIs, this work proposes a model of territorial analysis that serves to make decisions in situations where there are different environmental, social, political and economic contexts. Specifically, a classification of the municipalities of Andalusia in relation to the conservation status of their GIs is addressed which is subsequently related to their socioeconomic situation based on the selection of a set of indicators.

Based on the results obtained, among the public management recommendations, it is essential, first of all, to adopt a more sustainable model for the coastal strip and metropolitan areas of Andalusia. To this end, the quality of life of the people must be prioritized over the occupation and urbanization of land. This means that it is essential to recover and restore the GIs and to have healthier urban and agricultural spaces, where sustainable mobility models prevail, less water and energy are consumed, more is recycled and local and local commerce is promoted.

Public Authorities must commit to a new water culture throughout the region. For this, reversing the processes of the commodification and speculation of water must be prioritized, involving the users themselves in the control of illegal extractions; and promoting the recovery of aquifers in poor condition through reuse and desalination with renewable energy. In this sense, it is necessary to regenerate and reuse 100% the purified water and reach zero discharge to the hydraulic, and other land or maritime infrastructures which are publicly managed.

The ecosystem services provided by the region's GI are closely related to the persistence of traditional ecologically based land uses, which are in sharp decline as a consequence of the loss of population in inland and mountainous areas. Consequently, public financing mechanisms in the region must take into account these demographic imbalances, as well as possible compensations for the provision of ecosystem services, which must be aimed at guaranteeing equal opportunities and shielding essential public services in these rural zones. In this sense, the work already carried out by the regional government in recent decades in Protected Natural Areas aimed at promoting green employment, rural tourism, and ecology agriculture and livestock can serve as the foundation on which to base this new territorial model. Indeed, it is evident that these initiatives have provided a certain dynamism in the local economies of the Andalusian municipalities founded on sustainability criteria.

As a final conclusion, it can be affirmed that the municipalities in rural areas are “ecologically” financing the entire Andalusian population. Faced with this situation, the decisions and actions of policymakers in this region should promote measures aimed at restoring and conserving GI, addressing the demographic and/or socioeconomic imbalances of the region.

A limitation of this work consists in the exclusion of indicators related to specific resources (physical and monetary) and to the financial sustainability of public actions aimed at improving GI. In this sense, as a future line of research, it would be interesting to incorporate efficiency indicators of GI, which would report on those policies that allow the consumption of public resources to be minimized and have certain impact on environmental, social, and economic indicators.

## Data Availability

The datasets used and/or analysed during the current study are available from the corresponding author on reasonable request.

## Abbreviations

GI: Green Infrastructure
GIS: Geographical Information System
INE: National Statistics Institute
REDIAM: Environmental Information Network of Andalusia
SAC: Special area of conservation
SCI: Site of community importance
SPAB: Special protected areas under Bird Directive
